# Adaptive explanations for sensitive windows in development

**DOI:** 10.1186/1742-9994-12-S1-S3

**Published:** 2015-08-24

**Authors:** Tim W Fawcett, Willem E Frankenhuis

**Affiliations:** 1 Modelling Animal Decisions (MAD) Group, School of Biological Sciences, University of Bristol, Life Sciences Building, 24 Tyndall Avenue, Bristol BS8 1TQ, UK; 2 Behavioural Science Institute, Radboud University Nijmegen, Montessorilaan 3, PO Box 9104, 6500 HE, Nijmegen, The Netherlands

**Keywords:** Adaptive developmental plasticity, Autocorrelation, Bayesian updating, Behavioural consistency, Cue reliability, Critical period, Environmental predictability, Social behaviour, Value of information

## Abstract

Development in many organisms appears to show evidence of sensitive windows—periods or stages in ontogeny in which individual experience has a particularly strong influence on the phenotype (compared to other periods or stages). Despite great interest in sensitive windows from both fundamental and applied perspectives, the functional (adaptive) reasons why they have evolved are unclear. Here we outline a conceptual framework for understanding when natural selection should favour changes in plasticity across development. Our approach builds on previous theory on the evolution of phenotypic plasticity, which relates individual and population differences in plasticity to two factors: the degree of uncertainty about the environmental conditions and the extent to which experiences during development (‘cues’) provide information about those conditions. We argue that systematic variation in these two factors often occurs within the lifetime of a single individual, which will select for developmental changes in plasticity. Of central importance is how informational properties of the environment interact with the life history of the organism. Phenotypes may be more or less sensitive to environmental cues at different points in development because of systematic changes in (i) the frequency of cues, (ii) the informativeness of cues, (iii) the fitness benefits of information and/or (iv) the constraints on plasticity. In relatively stable environments, a sensible null expectation is that plasticity will gradually decline with age as the developing individual gathers information. We review recent models on the evolution of developmental changes in plasticity and explain how they fit into our conceptual framework. Our aim is to encourage an adaptive perspective on sensitive windows in development.

## Introduction

Phenotypes result from an interaction between evolutionary and developmental processes of adaptation: trait expression is adjusted during development in response to information about the environment, via molecular, physiological and psychological mechanisms that have evolved through natural selection [1,2]. This adaptive developmental plasticity often varies across the lifespan, punctuated by one or more *sensitive windows *(see glossary, Table 1) during which the phenotype is particularly responsive to environmental conditions. For example, social interactions early in life can have pronounced and lasting effects on behaviour [3-5], as highlighted by the pioneering studies on filial and sexual imprinting by ethologists such as Lorenz [6], Immelmann [7] and Bateson [8]. More recent work has identified adolescence as another sensitive developmental phase in many species for adapting to the social environment [9-18] (but see [19]), perhaps foreshadowed in humans by a developmental switch point in middle childhood [20]. Yet despite broad interest in these patterns, we lack a general understanding of why, from an evolutionary perspective, such sensitive windows in development exist.

**Table 1:** Glossary of technical terms used in this article

Term	Definition
*Autocorrelation*	A statistical association between environmental states across space or time. Positive temporal autocorrelation implies that conditions at one point in time are similar to those in the near future.
*Bayesian updating*	A method for revising a belief about the world in the light of new evidence, based on Bayes's rule. See Appendix A (additional file 1) for more details.
*Estimate*	A probability distribution for possible states of the world, based on the information available to an individual from its past experiences and evolutionary history. Note that no cognitive process, conscious or otherwise, is implied.
*Cue*	An experience that potentially provides information about environmental conditions. Note that cues can be uninformative, unreliable or even misleading (cf. information, which by definition is always informative).
*Information*	A reduction in uncertainty (about the state of the world).
*Informativeness*	The extent to which a cue reduces uncertainty.
*Mutual information*	The amount by which uncertainty is reduced (by observation of a new cue).
*Plasticity*	The degree to which cues received during development affect an organism's phenotype. Here we include both activational (or contextual) plasticity, whereby the organism immediately adjusts its phenotype in response to current cues, and narrow-sense developmental plasticity, in which there is a lasting phenotypic response to cues received in the past [21-23].
*Posterior*	In Bayesian updating, a revised estimate of the state of the world after new evidence has been taken into account.
*Prior*	In Bayesian updating, an initial estimate of the state of the world before new evidence is taken into account.
*Reliability*	The extent to which a cue indicates the true state of the world (either now or in the future).
*Sensitive window*	A developmental period or stage in which experience shapes phenotypic development to a larger extent than in other periods or stages. This definition encompasses both sensitive periods—in which plasticity is a function of chronological age—and sensitive stages—in which plasticity is not tied to a specific age but is a function of the organism's developmental stage, which can depend on its previous experiences (e.g. the duration of a sensitive window might depend on the consistency of experiences earlier in development [35]).
*Uncertainty*	The probabilistic nature of an organism's knowledge about the world, determined by factors beyond its immediate control but potentially reducible by sampling [94].
*Value of information*	The change in expected future reproductive success associated with a reduction in uncertainty. Note that this is always non-negative: information, once received, never reduces fitness [41].

To help address this problem, here we outline an adaptive framework for understanding when natural selection should favour changes in plasticity across development. We define plasticity as *the degree to which cues received during development affect an organism's phenotype. *This broad definition encompasses *activational plasticity * (an immediate phenotypic change in response to current cues; also termed *contextual plasticity*) as well as narrow-sense *developmental plasticity *(a lasting phenotypic change in response to cues received in the past) [21-23], both of which should be sculpted by the same fundamental selective pressures we identify below. Note that we are interested specifically in evolved patterns of plasticity (i.e. *potential plasticity *rather than *realised plasticity *[23]). We focus primarily on the development of social behaviour—that is, interactions with other (conspecific) individuals—but many of the points we make are equally applicable to other phenotypic traits.

**Figure 1:**
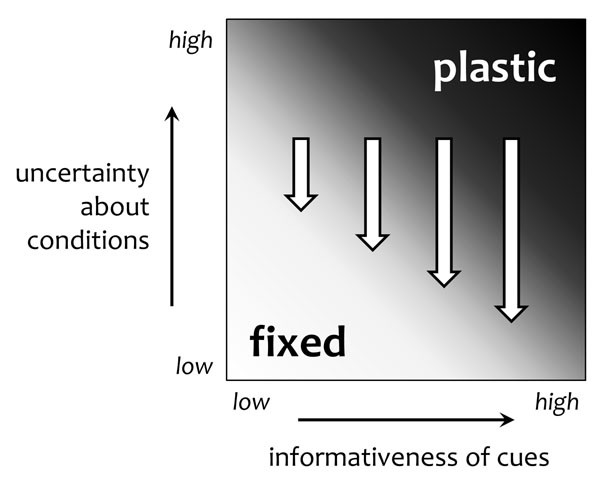
**Selection for fixed versus plastic phenotypes.** This schematic representation (inspired by [28,30]) shows how evolved plasticity should depend on uncertainty about environmental conditions and the informativeness of cues received during development. Plasticity (dark shading) is favoured when high uncertainty about environmental conditions is combined with cues that are highly informative about those conditions; otherwise, a fixed phenotype is favoured. Note that both of these factors (uncertainty about conditions and informativeness of cues) may change during ontogeny. For example, uncertainty will tend to decrease over development (as indicated by the white block arrows), particularly if the cues received are highly informative; this will weaken the benefits of plasticity later in life (all else being equal).

To set the stage for our framework, we briefly discuss *uncertainty *about environmental conditions and *informativeness *of cues during development (Fig. 1) as the major factors that drive plasticity differences between populations and between individuals in the same population. We then connect this work to patterns of development, by showing how the same perspective can be used to study adaptive variation in plasticity *within the lifetime of a single individual*. The key point we wish to make is that to understand adaptive developmental plasticity, we need to consider how informational properties of the environment interact with the life history of the organism [24]. Our aim is to stimulate an evolutionary, fitness-based approach to the study of sensitive windows in development.

## Review

### Adaptive explanations for variation in plasticity between and within populations

‘Darwinian demons’ , which can maximise all aspects of fitness simultaneously [25] and develop optimal phenotypes in all environmental conditions at any life stage [26], are rare or non-existent in nature. Organisms do typically tailor their phenotype to local environmental conditions [27], but the extent to which they do so varies between populations, within populations and within individuals over time. What ecological factors underlie this variation in plasticity?

#### Variation in plasticity between populations

Natural selection favours plasticity if organisms are uncertain about the present or future environmental conditions, and cues received during development provide information (i.e. reduce uncertainty) about those conditions [28,30] (Fig. 1). Organisms face uncertainty if environmental conditions fluctuate, in a stochastic manner, on timescales too fast for genetic evolution to track. If, alternatively, conditions are constant or change very slowly relative to the generation time, or if changes follow a highly regular pattern across the organism's lifespan (e.g. all individuals are born in the spring and reproduce in the autumn, when conditions are different), then organisms should evolve fixed phenotypes suited to those conditions (‘adaptive tracking’ by genetic evolution [30]). A study by Carroll and Corneli [31] on soapberry bugs (*Jadera haematoloma*) illustrates that selection for plasticity depends on the stochastic variability of environmental conditions over evolutionary time. In Oklahoma, where sex ratios are stochastically variable across generations (due to climatic fluctuations), males are plastic, calibrating the amount of mate guarding they perform to the sex ratio they have experienced in their own lifetime. By contrast, in Florida, where sex ratios are more stable, males engage in a fixed amount of mate guarding and, when exposed to variable sex ratios in the laboratory, are incapable of calibrating [31]. Thus plasticity has only evolved in those populations that are naturally exposed to stochastic variation in the sex ratio, such that individuals are born uncertain about the intensity of competition for mates.

Uncertainty does not by itself select for plasticity. For plasticity to be favoured, cues received during development must provide information about the present or future environmental conditions (Fig. 1) [29]. In Oklahoma soapberry bugs [31], for example, plasticity in mate-guarding behaviour is adaptive because the number of males and females an individual encounters provides information about the local sex ratio, and hence the competition for mates. Organisms may use cues during development to infer the present conditions, and if environmental states are highly autocorrelated over time then they might use those cues to predict future conditions as well [32,33]. If organisms cannot anticipate the environmental conditions due to lack of information about the present, weak autocorrelation with the future, or both, then natural selection might favour bet-hedging instead of plasticity [30,34].

#### Variation in plasticity within populations

Plasticity varies not only between populations (e.g. [31]) but also between individuals in the same population [35-37]. From an adaptive perspective, such differences may again be related to uncertainty about environmental conditions.

Between-individual differences may be fixed from birth or gradually emerge due to variation in early experience. Considering fixed differences, models have shown that stable between-individual variation in plasticity can be maintained in a single population through negative frequency-dependent selection [38] or parental bet-hedging [34]. Wolf *et al. *[38] modelled a scenario in which individuals living in an uncertain environment were either ‘responsive’ , in that they could assess (at a cost) the environmental conditions and adjust their behaviour accordingly, or ‘unresponsive’ , in which case they adopted the same behavioural strategy regardless of current conditions. At equilibrium the population comprised a mixture of responsive and unresponsive types, because the pay-off to each type was inversely related to its relative frequency (see also [39]). Using similar logic, Frankenhuis *et al. *[34] modelled offspring production in a stochastically varying environment. Parents could choose to invest in either plastic (‘generalist’ ) offspring that could adjust (at a cost) their phenotype to match current conditions, fixed (‘specialist’ ) offspring that were inflexible and only gained a high fitness pay-off if the current conditions happened (by chance) to match their predetermined phenotype, or a mixture of the two types. Given large fitness effects, greater costs of being mismatched than benefits of being well-matched and a temporally fluctuating environment in which conditions could change from one generation to the next, the most successful strategy was a form of diversified bet-hedging in which parents produced a mixture of plastic and fixed offspring [34].

Rather than being an intrinsic, stable characteristic fixed from birth, an individual's degree of plasticity could also be contingent on its early experience. Individuals that received a highly consistent set of cues early in life may later show reduced plasticity compared to those that received contradictory cues, because the latter remain more uncertain about current conditions and hence delay phenotypic commitments [35,36]; in such a case, the duration of a sensitive window is itself plastic. We return to this point below when discussing endogenous changes in information state.

### Adaptive explanations for variation in plasticity within the lifetime of a single individual

The above perspective suggests that phenotypic plasticity can be understood as an adaptive response to environments in which conditions are uncertain and cues received during development provide information about those conditions [28,30]. Building on this insight, our aim is to identify evolutionary reasons why phenotypes may be more or less plastic in different phases of development. We are specifically interested in situations where natural selection has generated changes in plasticity over the course of development, such that the same experience has differing phenotypic effects depending on the developmental period or stage at which it occurs. Central to our approach is the distinction between *information*—a reduction in uncertainty—and *cues*, the events during development that (potentially) provide the information [40,41].

From an adaptive perspective, changes in plasticity over the course of development reflect changes in the costs and/or benefits of being plastic, where costs and benefits are defined in terms of decreases and increases in expected future reproductive success. Here we identify four non-mutually-exclusive reasons why this might be the case, drawing on the distinction between cues and information. Selection will tend to favour changes in plasticity if there is systematic (i.e. partly predictable) variation across development in (i) the frequency of cues, (ii) the informativeness of cues, (iii) the fitness benefits of information and/or (iv) the constraints on plasticity. Of critical importance is how such variation coincides with particular phases of the organism's development, as determined by its life history. Below we discuss each of these factors in turn.

#### (i) Variation in the frequency of cues

The benefits of plasticity will change across development if, within lifetimes, there is systematic variation in the frequency of cues received by the organism that indicate the conditions relevant to that particular aspect of the phenotype. This frequency may vary because of changes in sampling (e.g. exploration), which is often an active process that is itself under selection [35,42], or because of changes in the availability of cues in the external environment. Assuming that processing of cues is not cost-free (see *(iv) *below), we would expect evolved patterns of developmental plasticity to anticipate this variation: individuals should show reduced plasticity during phases of development when cues are usually rare and heightened plasticity when cues are most frequent, all else (e.g. cue informativeness—see *(ii) * below) being equal.

Thus, if individuals are likely to encounter a new source of cues in particular phases of development, we would expect to observe an associated increase in plasticity. New cues may become available because individuals begin interacting with a completely new set of partners, for example after dispersal to a different social group. Early experiences in this new situation may have a formative impact on the phenotype. Alternatively, the interaction partners may stay the same but the nature of those interactions may change; for example, interactions with opposite-sex peers before puberty may provide very different cues (e.g. relating to social alliances or friendships) from interactions with those same peers after puberty (e.g. indicating potential reproductive opportunities). A third possibility is that both the type of social interactions and the interaction partners change simultaneously. In many species, independence from parents is marked by a shift from predominantly asymmetrical interactions with those parents to more symmetrical interactions with similar-aged peers, which may be one explanation why adolescence could be a sensitive phase for the development of social behaviour [16,43].

If the timing of such changes in an organism's social situation is variable, associated increases in plasticity need not be tied to specific time periods in development. Instead, selection may favour heightened plasticity when individuals are exposed to novel social situations and thus encounter new cues, regardless of the period of development in which this occurs. In species with very labile social systems, a high degree of plasticity may need to be maintained throughout life. For example, in the cichlid fish *Astatotilapia burtoni*, which has a highly dynamic social structure, males of all ages show striking changes in behaviour and reproductive physiology within minutes of a change in the composition of their social group [44,45].

#### (ii) Variation in the informativeness of cues

Even if cue frequency is constant throughout development, there may be variation in the informativeness of those cues, in terms of how much they reduce the receiver's uncertainty. This uncertainty reduction is what information theorists refer to as *mutual information*; note that it is distinct from the usefulness (e.g. fitness value; see *(iii) * below) of that information [41,46,47]. Systematic variation across development in cue informativeness could arise from exogenous or endogenous factors.

**Exogenous changes in cue reliability.** Cue *reliability *[23,28,48] (sometimes termed *validity *[35])—the extent to which cues reflect the true conditions—may change across development in a predictable way; this is an exogenous factor in the sense that it is not caused by changes in the organism perceiving those cues. If the environment is variable and autocorrelated over time, then cues indicating a situation that will be experienced in the future will typically be more reliable closer to that point in time [32]. For example, cues indicating the intensity of mating competition an individual can expect to face as an adult will tend to be more reliable in late adolescence than in early childhood. This may promote heightened plasticity as sexual maturity approaches.

Very early in development much of the available information is transgenerational, which has interesting implications for cue reliability. Maternally derived cues in the prenatal environment may more reliably predict future external conditions than those experienced directly by the offspring after birth or hatching, because the mother's greater exposure to external conditions provides a more informative cue. From this perspective, the mother is viewed as an experienced integrator of cues who can filter out useful information from background noise [49,50], which may partly explain why the prenatal environment has such a lasting impact on development in many species [5,51-56]. On the other hand, the need for the mother to provide a protective environment, which buffers her developing offspring against more variable and potentially damaging conditions in the external environment [57], may limit the informativeness of conditions *in utero*. A recent meta-analysis of experimental studies revealed limited evidence that transgenerational plasticity confers fitness benefits on offspring [58], perhaps because of weak correlations between parent and offspring environments [29].

The reliability of maternal cues in forecasting future conditions for offspring is strongly dependent on the species’ ecology and life history. Maternal cues may be informative either because adults and offspring occupy similar ecological niches, or because the mother's own experiences as a juvenile reliably predict her offspring's juvenile environment. In the cichlid *Simochromis pleurospilus*, juveniles and adults occupy different habitats with associated differences in food availability and predation risk, which render the mother's adult environment a poor predictor of the conditions her offspring will experience when they reach independence [59,60]. Consequently, mothers tailor their investment in eggs in response to the food availability they themselves experienced as juveniles [59], whereas maternal effects on antipredator behaviour are quickly overridden by the offspring's own experiences [60].

In general, the benefits of plasticity will be affected by how reliably current cues predict environmental conditions at the target life stage(s) at which selection acts. Correlation between current and future conditions is thus critical. Correlograms—plots of the temporal autocorrelation over different time lags—can be a useful tool in certain study systems for assessing the predictability of conditions across development [29].

**Endogenous changes in information state.** Endogenous factors reflect how information is accumulated over the course of development. Even when confronted with equally reliable cues, individuals at different developmental stages will often differ in their uncertainty about environmental conditions, leading to different levels of sensitivity to new information. Typically, older individuals will have been exposed to more cues than younger individuals and so, providing those cues were informative, they will have lower uncertainty about the true state of the world. This implies that they will be less influenced by new cues than are individuals at earlier stages of development.

We can use a Bayesian approach (see Appendix A, additional file 1) to examine how sensitivity to new cues should change as individuals become better informed over the course of development. We first discuss a case where environmental conditions remain constant throughout development, before considering a situation in which conditions vary, with some degree of predictability, across ontogeny. For illustrative purposes, consider a simplified scenario in which an individual is uncertain which of two situations it faces, but the appropriate behaviour differs between the two situations. For example, continuing with the soapberry bug example mentioned above [31], a male might not know whether the competition for mates is high or low, but mate guarding is only adaptive in the former situation. We can conceptualise the male's current estimate (at time *t*) regarding its social circumstances as a probability *p_t_*, with *p_t_* = 0 representing certainty about one situation (e.g. that there is low competition for mates, due to an excess of females in the adult population) and *p_t_* = 1 representing certainty about the alternative situation (e.g. that there is high competition for mates, due to an excess of males in the adult population). Uncertainty is greatest at *p_t_* = 0.5, where the male estimates (non-consciously) that both situations are equally likely. Note that when discussing what an individual ‘estimates’ or ‘knows’, we are not referring to any particular cognitive processes; this is merely a convenient shorthand to capture the information available to the individual, based on a combination of its past experiences and evolutionary history. In general, we expect that natural selection will have produced animals that behave *as if* they knew the probabilities associated with different situations [61,62].

**Figure 2:**
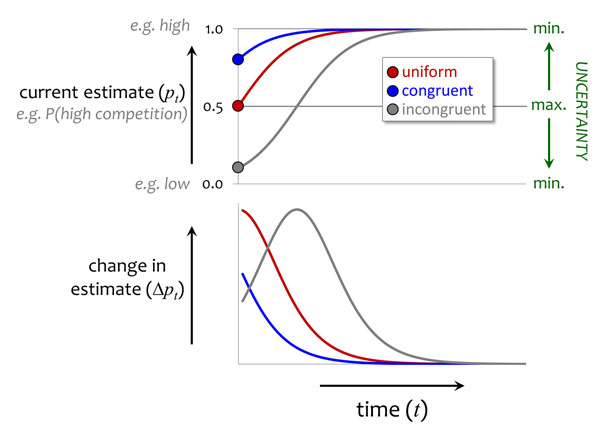
**Developmental changes in information state when conditions are stable.** The upper panel shows the optimal estimate (*p_t_*) of the environmental state in response to a steady stream of information (from cues of fixed reliability), calculated using Bayesian updating (Appendix A, additional file 1). The lower panel shows the extent to which the estimate changes (Δ*p_t_*) when a new cue is observed. The three different lines depict the estimates for individuals in three cases, depending on whether the initial estimate is a uniform prior (i.e. both states are equally likely), is in agreement with subsequent cues (‘congruent’) or is contradicted by subsequent cues (‘incongruent’ ). Note that sensitivity to environmental cues declines with time, unless the observed cues contradict the initial estimate. See text for more details.

Now imagine that, during development, the individual receives a steady stream of information about its circumstances (Fig. 2). Specifically, we assume that new cues are detected at a constant rate, so there is no variation in cue frequency (see *(i)* above), and that all cues are equally reliable, so we can also rule out *exogenous* variation in cue informativeness (see above, this section). In our example, this corresponds to the male soapberry bug encountering adult conspecifics at a constant rate, and having a fixed ability to detect their sex. If, at the start of development (*t* = 0), the individual estimates the two situations to be equally likely (i.e. it has a uniform prior, *p*_0_ = 0.5), then cues received early in the sequence will reduce its uncertainty to a greater extent (in other words, will be more *informative*) than those received later on (Fig. 2, red line). This is because at later stages in development the individual has received more information, and so has greater certainty about which particular situation it faces. The same applies when the information it receives during development is congruent with (and thus strengthens) its initial estimate about which situation is more likely (Fig. 2, blue line).

A different pattern of sensitivity arises if information received during development contradicts the initial estimate; that is, if developmental experiences cause *p_t_* to increase from an initial estimate of *p*_0_< 0.5 (as shown in Fig. 2, grey line), or equivalently if they cause *p_t_* to decrease from *p*_0_> 0.5. In this case, the extent to which new cues affect the current estimate rises and then falls over the course of development, such that the period of greatest sensitivity occurs at an intermediate age. In general, peak sensitivity to new information coincides with the point in development when uncertainty is greatest, i.e. when the probabilities of the different possible states of the world are most similar (in this simple case of two alternative states, *p_t_* = 1 − *p_t_* = 0.5). In most cases this will be at the start of development, but as the ‘incongruent’ case shows (Fig. 2, grey line), it is theoretically possible, at least in a scenario with only two possible states of the world, for uncertainty to be greatest later in development.

What determines the initial estimate (*p*_0_)? This could represent inherited genetic information, reflecting the relative exposure of the focal individual's ancestors to different types of conditions in the past [61], or perhaps non-genetic parental effects, such as maternal effects set by the specific experiences of the focal individual's mother [63]. Typically (unless conditions have changed suddenly and unpredictably) this prior information will be an accurate estimate of the likelihood of each possible situation the individual might face, otherwise the organism should have evolved to ignore it. Therefore, the prior information is likely to be congruent with cues received subsequently during development. This is one reason why developmental experiences that occur early in life will—all else being equal—tend to have the most powerful impact on the phenotype. Thus, when considering developmental changes in plasticity, a sensible null expectation is not that plasticity remains constant, but that it declines with age. We might therefore predict that the rate of change in behaviour is higher earlier in ontogeny and that individuals should show greater consistency in their behaviour as they mature and gain experience [48], a pattern for which there is some empirical evidence [48,64,65]. This declining sensitivity to environmental cues over the course of development neatly parallels the evolutionary prediction that reduced uncertainty favours a less plastic phenotype [28,30,66], as indicated in Fig. 1.

So far, for illustrative purposes, we have considered a highly simplified scenario in which there are only two possible states of the world. In reality, the relevant environmental conditions will often show continuous variation, or at least a large number of possible states—for example, the optimal level of mate-guarding behaviour might depend not merely on whether adult males outnumber adult females, but on the precise sex ratio. This alters the dynamics of information, because unlike in a two-state scenario the organism's current estimate of the environment and its uncertainty regarding that estimate can change independently (see Appendix A, additional file 1). A recent model by Stamps and Krishnan [48] considered a large number of possible states, rather than just two, to investigate how developmental trajectories differ when individuals with different initial estimates are given the same set of cues. Their model predicted that the change in uncertainty is always greatest at the start of development regardless of the initial estimate, even if subsequent cues contradict that estimate. Thus, the expected decline in plasticity over ontogeny may be a more robust prediction than a simple two-state model suggests. A delayed peak in plasticity (grey line, Fig. 2) is possible in principle, but it appears to occur only in the two-state model, and then only in the unlikely case that the cues received during development contradict the organism's initial estimate.

There is an important caveat to these predictions. Although the optimal phenotype should reflect the animal's underlying information state [48,62,67], this phenotype might be biased towards situations associated with higher fitness returns [68]. Consider aggression in the face of uncertainty; an individual may not know whether it is stronger or weaker than its rivals, but this information is important for adopting an appropriate level of aggressive behaviour [35,36,69]. If the fitness returns (when following the optimal strategy) are much higher for an individual who is stronger than its rivals than one who is weaker than its rivals, it pays to start out behaving as if the former is true, even if this is statistically unlikely to be the case [69]. Such a phenotypic bias can cause a lag between the (unobservable) change in information state and the (observable) change in behaviour, leading to a delay in the period of peak plasticity. Only in cases where similar fitness can be achieved under all environmental conditions would we expect a direct correspondence between changes in information state and observable phenotypic changes.

**Figure 3:**
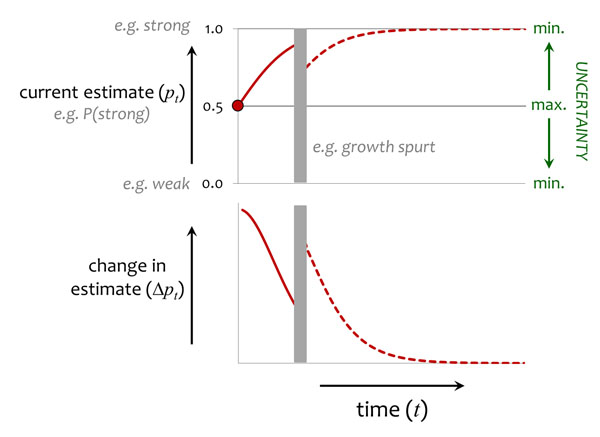
**Developmental changes in information state when conditions change.** As for Fig. 2, except that uncertainty increases mid-way through development because individuals encounter a new social situation. For clarity, only the trajectory with the uniform prior is shown. See text for more details.

The above logic applies to cases in which conditions are reasonably stable across ontogeny. Trajectories of plasticity may be different if conditions can change during development [26]. Rather than showing a gradual decline, uncertainty may suddenly increase when individuals are faced with a new social situation, because of ecological factors (e.g. dispersal to a new social group) or physical development (e.g. rapid increase in muscularity). For example, periods of physical development that are highly variable between individuals in their extent or timing may generate renewed uncertainty about relative strength part way through development, leading to a second phase of heightened sensitivity to new social experiences (Fig. 3). The adolescent growth spurt in humans may have this kind of effect.

In the most dynamic social systems, it may be advantageous to retain a high degree of plasticity throughout life. This appears to be the case for the cichlid *A.burtoni*, in which reversals of social dominance between males can happen at any time [70-72]. Dominant males are not only more vulnerable to predation (because of their greater conspicuousness) but also grow more slowly than subordinates, so ‘overtaking’ in growth is common [72]. This makes the dominance hierarchy unstable, promoting high plasticity throughout life in this species [44,45] because uncertainty about future conditions remains high. Interestingly, brain and hormonal changes occur more rapidly in males ascending the hierarchy than descending it [73], a bias that may be favoured by the fitness asymmetry between breeding and non-breeding positions (i.e. reproductive skew).

Within a population, individual differences in plasticity may emerge in relation to the consistency of cue sets received earlier in development, because of how this affects information state. Uncertainty about the future environmental state will typically be greater for individuals that experience stochastically changing conditions than those that experience stable conditions [26]. Thus, if maintaining plasticity is costly [74,75], individuals that have had highly variable experiences early in life should retain a greater degree of plasticity than those exposed to relatively stable conditions [36]. In effect, the pattern of plasticity may itself be plastic.

This phenomenon is well established for sensory systems: individuals experiencing a transient shift in their sensory environment show greater neuronal plasticity when the same experience is repeated later in life, compared to those experiencing the change for the first time [76,77]. At a behavioural level, Kotrschal and Taborsky [78] showed that in *S. pleurospilus*, a mouth brooding cichlid, adults perform better in an associative learning task if they experienced a change in food availability as juveniles, regardless of the direction of the change (increase or decrease). In this setting, experiences early in life appear to be used as a source of information about the temporal stability of environmental conditions.

Uncertainty about environmental conditions will also be greater for individuals that receive noisy or error-prone cues, which again may promote plasticity [35,36,79]. For example, auditory specialisation in rats (*Rattus norvegicus*) reared with a continuous background of moderate-intensity white noise is delayed well beyond the critical window observed for rats reared under standard laboratory conditions [80]. The same principle should apply to noisy internal neural signals as well as noisy external environmental cues. An interesting possibility is that humans suffering from certain developmental disorders, such as autism spectrum disorder, prolong plasticity as an adaptive response to atypically high levels of error in neurally encoded information [79]. Although the noisy neural signals associated with autism are themselves probably maladaptive (in evolutionary terms), keeping sensitive windows open for longer may represent the best way of coping with this impaired state.

It should be clear from the various scenarios discussed above that an individual's response to new information is strongly dependent on its prior information state, which can generate a variety of patterns of plasticity across development. The essential point we wish to make is that, even with no changes in the frequency or reliability of cues, it is inevitable that cue *informativeness *will vary across development, so we should not expect individuals to be equally plastic at all ages or developmental stages. In social environments that are reasonably stable within an individual's lifetime, our null expectation should be that, all else being equal, plasticity declines across development.

#### (iii) Variation in the fitness benefits of information

Even controlling for variation in the frequency and informativeness of cues, there may be additional variation in the fitness benefits of information received across development—that is, the same amount of information may be more or less valuable at different stages of life. Once acquired, information never reduces fitness [41]. However, given the same amount of information (i.e. the same reduction in uncertainty), the increase in expected future reproductive success from making phenotypic adjustments in response to this information often varies across development. One general point is that phenotypic adjustments late in life will typically have a lower impact on fitness than those early in life, because fewer individuals survive to older ages and thus the force of selection declines with age [81-83].

In specific contexts there may be additional sources of variation in the fitness benefits of plasticity, depending on the ecological and life-history characteristics relevant to that aspect of the phenotype. For example, in the context of mutual mate choice, it is adaptive for individuals to modify their choice strategy in response to social feedback about their own attractiveness [84,85]. However, individuals approaching the end of their reproductive lifespan will gain relatively little benefit from adjusting their mate-choice behaviour and so should be less sensitive to social feedback than those with most of their reproduction still ahead of them. Individuals might also differ in sensitivity to social feedback depending on their current relationship status (e.g. whether they are settled in a long-term relationship, are considering ending such a relationship, or are single). Taking filial imprinting as another example, there are likely to be strong benefits of following a familiar stimulus (i.e. the parent) around when an individual is small, naïve and vulnerable to predation [8], but much less so when it is bigger, experienced and able to defend itself.

#### (iv) Variation in the constraints on plasticity

If there were no constraints on plasticity then individuals should evolve to be maximally plastic all the time [74], even if cues are sparse or barely informative. To understand patterns of adaptive developmental plasticity, we therefore need to consider the constraints as well as the potential benefits [41].

DeWitt *et al. *[74] distinguished between two broad classes of constraint: limits and costs (but see [86]). Plasticity is *limited* if plastic development cannot achieve the same range of phenotypes as fixed modes of development. For example, the time or resources required for phenotypic adjustments may mean that the range of possible phenotypes is more restricted as development progresses, making it increasingly difficult to switch to an alternative developmental trajectory [35,36,53,87,88]. The limits could also vary across development if there are particular periods in which plasticity trades off with other traits that are important at the same life stage. For instance, plasticity genes could have detrimental pleiotropic effects on traits other than the focal plastic trait, and these effects might be stronger in some life stages (e.g. during organogenesis) than others [74]. Accordingly, even if the frequency and informativeness of cues remains constant, the ability to use that information may vary across ontogeny.

Plasticity is *costly* if a plastic individual can produce the same phenotype as one with a fixed developmental pattern, but by doing so it has lower fitness [75,86]. Here we briefly mention three main types of cost (see also [74,75]):

1. *Start-up costs: *the energy and/or resources to build and maintain the neural-cognitive machinery required for plasticity. For example, in selected lines of *Drosophila*, larvae of flies with higher learning ability showed reduced survival under competition, demonstrating a fitness cost of this aspect of plasticity [89].

2. *Running costs: *the energy and/or resources to obtain information and process phenotypic changes. Note that these costs are only paid when the machinery for plasticity is actually used, distinguishing them from start-up costs (see 1 above). Evidence for running costs also comes from studies of *Drosophila*: the formation and maintenance of long-term memory appears to reduce resistance to starvation and desiccation in adult flies [90].

3. *Error costs: *the fitness costs of ‘getting it wrong’ . Individuals might develop an inappropriate phenotype if they misperceive reliable cues, if they accurately perceive cues but incorrectly execute actions, or if they respond to irrelevant cues as though they were informative. As an example of the latter, consider sexual imprinting in obligate brood parasites [91]. The machinery for sexual imprinting should be actively switched off while the parasite offspring is still in the host nest, otherwise it will end up imprinting on the wrong species. Imprinting sexually on its nest-mates would actually be harmful to the parasite's reproductive success (unless this imprinting could be completely overwritten by the first experience with conspecifics, which might not be possible due to other constraints).

### Synthesis with existing theory

A handful of recent mathematical models have explored various conditions under which evolution can lead to varying degrees of plasticity across development [26,35,48,88,92]. We now briefly describe each of these models and explain how they fit into our adaptive framework outlined above.

Frankenhuis and Panchanathan [35] modelled development as a constructive process, in which phenotypes incrementally adapt to local environmental conditions. They considered two environmental states (e.g. safe or dangerous) with different phenotypic optima (e.g. fast-moving versus heavily armoured). The environmental state remained stable throughout development but was unknown to the developing organism. The organism started ontogeny with an inherited prior estimate of the environmental state, which it could then update (in a Bayesian manner; see Appendix A, additional file 1) by sampling cues of fixed reliability. Crucially, sampling and phenotypic specialisation were assumed to be mutually exclusive activities; at each time step, the organism could either adjust its phenotype in a particular direction or sample an environmental cue. The degree of phenotype–environment match at the end of ontogeny (which occurred after a fixed number of time steps) determined fitness. The model predicted a sensitive period early in ontogeny, in which individuals sampled environmental cues before specialising towards the most appropriate phenotype given their information. Moreover, stochastic sampling led to individual differences in plasticity: individuals who experienced more consistent cue sets developed more confident estimates of the environmental state sooner and hence switched from sampling to specialisation earlier in ontogeny, losing their plasticity at faster rates. In terms of our conceptual framework, two factors explain changes in plasticity in this model (Table 2): endogenous changes in information state (estimates of the environmental state typically improve over ontogeny) and increasing limits to plasticity (fewer phenotypic adjustments are possible as the end of ontogeny approaches).

**Table 2:** Summary of five recent mathematical evolutionary models that predict variation in plasticity across development

	Factor(s) varying across development					
Model	Cue frequency?	Cue reliability?	Information state?	Benefit of information?	Limits to plasticity?	Cost of plasticity?
Frankenhuis and Panchanathan [35]	N	N	Y	N	Y	N
Panchanathan and Frankenhuis [88]	N	N	Y	N	Y	N
Stamps and Krishnan [48]	N	N	Y	N	N	N
English et al. [88]	N	N	Y	N	N	N
Fischer et al. [26]	N	N	Y	Y	N	N

Letters identify which factors did (Y) and did not (N) vary across development in each model. Factors varying across development could potentially explain the predicted developmental changes in plasticity.

In a follow-up model, Panchanathan and Frankenhuis [88] examined a scenario in which sampling and specialisation do not trade off; instead, individuals (passively) collect cues throughout ontogeny while building their phenotypes. This allows the organism to continue to change its mind while constructing its phenotype and to switch developmental trajectories accordingly. In this model, organisms not only accrued fitness benefits for correct phenotypic increments (i.e. phenotypes that matched the local environmental conditions), but also incurred fitness penalties for incorrect increments. Just like the earlier model [35], the new model predicted that organisms should become less sensitive to environmental cues over the course of ontogeny, and in some cases come to ignore them entirely. The more reliable the cues were, the faster plasticity declined. Again, stochastic sampling generated individual differences in sensitive windows: individuals who sampled more consistent cue sets lost their plasticity earlier in ontogeny. A novel prediction was that, in some cases, individuals should not switch their developmental trajectories even if they change their estimate about the most likely environmental state, because of an asymmetry in the associated fitness returns. Perseveration might be adaptive, for instance, if additional increments towards the original phenotypic goal (if correct) would yield higher fitness gains than initial increments towards the alternative phenotype (if correct), even if the former gains are less likely. Relating this model to our framework (Table 2), the same two factors explain changes in plasticity as in the model of Frankenhuis and Panchanathan [35]: endogenous changes in information state and increasing limits to plasticity.

Stamps and Krishnan [48] also modelled developmental trajectories over a fixed time period and with environmental cues of fixed reliability, but using 100 possible environmental states rather than just two. Again, conditions remained stable over ontogeny. The authors assumed that an individual's behavioural phenotype is directly tied to its information state and considered how this is influenced by different initial estimates (priors), reflecting genetic and non-genetic information received from ancestors at birth or hatching. The model predicted that behaviour changes most rapidly early in ontogeny, when individuals have least information about current environmental conditions. In terms of our framework, this heightened sensitivity to experience is explained by endogenous changes in information state (Table 2), with sensitivity declining gradually as individuals become better informed. The same endogenous changes in information state also led to an increase in the temporal consistency of behaviour over the course of development [48].

English *et al. *[92], like Frankenhuis and Panchanathan [35,88], studied optimal development in a world with two possible environmental states (food-rich and food-poor). Unlike other models [26,35,48,88], however, they allowed a flexible time period for development rather than assuming this to be fixed, and thus the constraints on plasticity did not change over ontogeny. English *et al. *explicitly modelled the process of growth, in which phenotypic adjustment and information gain are inextricably linked—a higher rate of food intake not only leads to increased body size, but also increases the posterior estimate (see Appendix A, additional file 1) that the environment is food-rich. This coupling sets it apart from other models in which phenotypic adjustment and information gain are assumed to be either mutually exclusive [35] or completely independent [26,88]. In the model by English *et al.*, individuals chose how much to forage (under predation risk) in each time step, which affected their probability of finding food, and at what body size to mature given their current estimate of the environmental conditions. The model predicted that foraging experiences early in life had a greater effect on foraging behaviour and the timing of maturation than similar experiences later in life, due to a decline in uncertainty with age (Table 2).

Finally, Fischer *et al. *[26] modelled optimal patterns of age-dependent plasticity in an environment where conditions could fluctuate over time. As in the models by Frankenhuis and Panchanathan [35,88] and English *et al. *[92] they considered just two environmental states, favouring alternative phenotypic specialisations. Like Panchanathan and Frankenhuis [88] they allowed sampling and specialisation to be independent: in a given time step, the developing individual could both sample environmental cues and adjust its phenotype. Phenotypic adjustment was assumed to be costly but not limited, in that the individual could potentially change its phenotype all the way from one extreme to the other within a single time step, but its survival and/or fecundity was reduced by an amount proportional to the degree of change. In contrast to the other models mentioned above the environmental conditions could change during the organism's lifespan, following a pattern of positive temporal autocorrelation. A maximum lifespan was imposed but reproductive success was accrued at every time step, according to the match between the organism's current phenotype and the current environmental conditions. The model predicted a peak in plasticity at the start of development, followed by a fat tail of moderate plasticity extending into later life if the probability of environmental change was sufficiently high. These patterns are explained primarily by endogenous changes in information state (Table 2): in a relatively stable environment the developing individual gradually becomes better informed, but when conditions are more changeable this generates additional uncertainty that can favour prolonged plasticity. Another contributing factor was that the value of information declined across the lifespan in the model, due to a mortality risk in each time step that discounted the value of future reproductive gains (Table 2).

The summary in Table 2 clearly shows that the focus of these adaptive models has been on endogenous changes in information state—a common factor to all the models is that individuals typically become better informed over the course of development (see also [93] for analogous effects in a model of language acquisition). Specific models have also incorporated declining fitness benefits of information [26] and increasing limits to plasticity [35,88]. To our knowledge, no models of adaptive developmental plasticity have addressed changes in the frequency of cues, exogenous changes in cue reliability or changing costs of plasticity as possible underlying factors. This would be a valuable direction for future work.

## Conclusions

### Key points and future directions

We have used an information-based perspective, focusing on uncertainty and informativeness as the key evolutionary drivers of plasticity, to identify adaptive reasons why individuals may be more or less sensitive to environmental cues at different points in development. Previous work has shown that plasticity is favoured when organisms are uncertain about the environmental conditions but can reduce that uncertainty through informative cues received during development. This offers a useful framework to understand differences in plasticity both between populations [28,31,66] and between individuals in a single population [23,36,48,78]. Here, we have used the same principles to address changes in plasticity across the lifetime of a single individual (Fig. 1). Broadly speaking, sensitive windows are more likely when organisms are uncertain about environmental conditions, receive many informative cues, are unconstrained in adjusting their phenotypes and fitness depends strongly on the phenotype–environment match.

Optimality models have highlighted endogenous changes in information state as an important adaptive explanation for changes in plasticity across development (Table 2). In relatively stable environments, if individuals face a more or less constant stream of information about the environmental conditions (i.e. constant frequency and reliability of cues), they should become increasingly certain about those conditions as they age and gain experience [35,48], which will favour a decline in plasticity across the lifespan. That is, all else being equal, phenotypic development should be most sensitive to experiences early in life. As we have discussed above, there are other factors—including ontogenetic changes in the benefits, costs and constraints associated with phenotypic adjustments—that will alter this pattern. However, we propose that an age-dependent decline in plasticity is the appropriate null expectation from an information-based perspective, and certainly a more sensible expectation than constant, age-independent plasticity. There is some empirical support for this pattern, in terms of increasing repeatability of behaviour with age [48,64,65]. In cases where plasticity follows a different developmental trajectory, and especially if there is evidence for heightened plasticity later in life, we encourage researchers to examine these patterns through the lens of our conceptual framework to identify underlying ecological or developmental factors.

Fundamentally, adaptive developmental plasticity is shaped by the interaction between the statistical properties of the environment (e.g. the timescale of variability in conditions, the degree of autocorrelation and the noisiness of environmental cues) and the organism's life history [24,29,32,60]. It is this interaction that determines both how uncertain the organism is about the environmental conditions at the relevant stage of its life, and to what extent its experiences during development reduce this uncertainty. For contexts in which the optimal phenotype depends on easily measurable properties of the physical environment (e.g. water temperature) it might be straightforward to characterise the statistical structure of the environment, or more specifically to obtain ‘quantitative estimates of environmental predictability in the field over the space and time scales relevant to the life history of the study organism’ ([29]). In principle these estimates can then be used to predict patterns of adaptive developmental plasticity.

For many other contexts, however, making predictions is likely to be more difficult. This is particularly the case for social aspects of the environment, such as the mixture of behavioural types in a population. Here, the interplay between the environmental structure and the organism's behavioural phenotype is complex, because the social environment both influences and arises from the behavioural strategies adopted by individuals in the population. For making clear predictions about how plasticity should vary across development in such social contexts, mathematical and computational models are invaluable tools. Existing models of adaptive developmental plasticity have taken an individual optimisation approach in which the environmental conditions are unaffected by the organism's phenotype [26,35,48,88,92]; developing related models for game-theoretical situations is a key challenge for the future. Another priority for theoretical work should be to examine how predictions derived from Bayesian models are constrained by their assumptions regarding environmental variation. Most models of adaptive developmental plasticity consider just two possible states of the world (e.g. [26,35,88,92], but the predictions from models allowing more than two states (e.g. [48]) may differ in important ways (see ‘Endogenous changes in information state’, above). Such differences require further attention.

We have identified several adaptive reasons why natural selection can result in developmental mechanisms that produce sensitive windows. Further work may reveal other possible explanations, adaptive or non-adaptive, that are important for understanding variation in plasticity across the lifespan. Ultimately, the field would benefit from a comprehensive framework specifying the different reasons why sensitive periods exist and their key predictions, which can then be tested empirically. Here we have sketched out parts of that framework. To the extent that development can be viewed as a process of ontogenetic adaptation, whereby organisms living in variable environments tailor their phenotypes to suit the particular conditions they encounter, an explicitly information-based perspective can yield important insights.

## Declarations

Publication costs for this article were funded by the German Research Foundation (FOR 1232) and the Open Access Publication Fund of Bielefeld and Muenster University.

## Competing interests

The authors declare that they have no competing interests.

## Authors’ contributions

TWF and WEF conceived and developed the ideas in this paper. TWF drafted the manuscript; both authors revised its content and approved the final version.

## Supplementary Material

Additional file 1: Appendix A Click here for file
