# Supplementary material for: Adaptive explanations for sensitive windows in development
Source: Front Zool. 2015 Aug 24;12(Suppl 1):S3. doi: 10.1186/1742-9994-12-S1-S3 (PMC4722342; doi:10.1186/1742-9994-12-S1-S3)
Supplement: Additional file 1: Appendix A [file 1742-9994-12-S1-S3-S1.docx]

Fawcett & Frankenhuis. Supplementary Material

**APPENDIX A**

**A Bayesian approach to information updating**

Bayes’s theorem, named after the Reverend Thomas Bayes, specifies how a subjective belief should change in the light of new evidence. Consider an individual that is uncertain about the state of the world. Before the new evidence arrives, its estimate can be represented by a probability, *p*(*s*), that the true state is *s*. The individual then perceives some cue, *c*, which perhaps represents a particular event or experience during its development. Its new estimate is then *p*(*s*|*c*), the conditional probability that the true state of the world is *s* having observed cue *c*. According to Bayes’s theorem this is given by

$p(s|c)=\frac{p(c|s)\cdot p(s)}{\sum_{s'} p(c|s')\cdot p(s')}$.

Here, *p*(*c*|*s*) is the *likelihood* of the cue, that is the conditional probability that cue *c* is observed given that the state of the world is *s*. The initial estimate *p*(*s*) is referred to as the *prior*; the revised estimate *p*(*s*|*c*) is referred to as the *posterior*. The product of the likelihood and the prior is divided by a normalising factor (i.e. the denominator on the right-hand side), which represents the overall probability of observing cue *c*, summed over all possible states of the world *s*′.

Bayes’s theorem can be used iteratively to calculate how the individual’s estimate changes when they observe a sequence of cues, with the posterior from one update becoming the prior for the next update. This process, referred to as Bayesian updating, is the theoretically optimal way to update estimates on the basis of past experience [48,61,62,95,96]. An essential first step is to describe the distribution of prior probabilities. The simplest situation (as illustrated in Fig. 2) is where there are just two possible states of the world, *A* and *B*, because then the priors *p*(*A*) and *p*(*B*) sum to 1. If there are many possible states of the world, however, the prior distribution can take on many different forms. For example, Stamps and Krishnan [48] modelled a scenario with 100 possible levels of predation risk, which required them to specify 100 prior probabilities and then update these at each iteration. Compared to a two-state model, the dynamics are more complicated because the mean value (representing the best estimate of the state of the world) is uncoupled from the variance (representing the uncertainty in that estimate); see online appendix A in [48]. A popular choice for Bayesian models is to assume a so-called beta distribution, because this ensures that the distribution of posterior probabilities will also be a beta distribution, which simplifies calculations. See [62] for further details.
